# Commencement and Monitoring: Microbiological Surveillance in Operation Theaters at a Tertiary Care Center in North India

**DOI:** 10.7759/cureus.58690

**Published:** 2024-04-21

**Authors:** Sana Islahi, Sweta Singh, Suyash Singh, Pragati Garg, Shefali Gupta

**Affiliations:** 1 Microbiology, All India Institute of Medical Sciences, Rae Bareli, Rae Bareli, IND; 2 Neurological Surgery, All India Institute of Medical Sciences, Rae Bareli, Rae Bareli, IND; 3 Ophthalmology, All India Institute of Medical Sciences, Rae Bareli, Rae Bareli, IND

**Keywords:** surface culture, settle plate method, microbiological surveillance, microbiology, pathological organisms, monitoring, commencement, operation theaters

## Abstract

Introduction: Preserving sterility and safety in hospital operation theaters (OTs) is vital. We have implemented a comprehensive microbiological surveillance program for OTs, encompassing both commencement and ongoing monitoring. This study assesses the prevalence of microorganisms, identifies their types, and detects contamination on surfaces and in the air.

Methods: Commencement and monitoring samples were collected from October 2021 to July 2023, from nine OTs. OTs were cleaned with soap and water, disinfected, and fogged with quaternary ammonium compounds. After sealing the OTs overnight, samples were collected aseptically. Air was sampled using the settle plate method, and surfaces were swabbed. Six surfaces, namely, the floor, wall, table, light, anesthesia workstation, and door handle, were swabbed. Samples were transported immediately to the institution's microbiology laboratory.

Results: During OT commencement, 247 swabs from nine OTs yielded 19 (7.29%) positives for bacterial growth. These microorganisms were primarily non-pathogenic, including aerobic spore-forming bacilli and Micrococcus, with an average bioload of 9.5 colony-forming units (CFU)/m^3^ of air. During OT monitoring, swab positivity was 10.79% (23/213). The General Surgery OT and Obstetrics and Gynecology OT showed the highest bacterial growth (5/23). Surface sampling revealed prevalent methicillin-resistant coagulase-negative staphylococci (MRCoNS) (9/23), followed by methicillin-sensitive *Staphylococcus aureus* (MSSA) (4/23) and methicillin-sensitive coagulase-negative staphylococci (MSCoNS) and aerobic spore-forming bacilli (ASB) (3/10). The General Surgery, Obstetrics and Gynecology, and ENT OTs displayed elevated air bioloads of 53, 49, and 47 CFU/m^3^, respectively.

Conclusion: In newly constructed non-operational OTs, non-pathogenic organisms prevailed. However, as the OTs became functional, pathogenic organisms became more prevalent. Sampling emphasized contamination in areas with high patient loads, such as General Surgery, Obstetrics and Gynecology, and ENT OTs. Notably, OT tables and OT walls exhibited higher pathogenic microorganism presence. By combining both initial commencement and ongoing monitoring, the institution has effectively managed the microbial environment within its OTs.

## Introduction

Surgical procedures, conducted within the controlled environment of operation theaters (OTs), represent a critical aspect of modern healthcare. The maintenance of sterility in OTs is of paramount importance to minimize the risk of surgical site infections and post-operative complications, which can significantly affect patient outcomes. In the wake of emerging healthcare challenges and the establishment of newly constructed tertiary care centers, the need for rigorous microbiological surveillance within these OTs is more pronounced than ever.

Microbiological surveillance is crucial in infection control programs, providing data on microbial flora types and counts [1]. Hospital-associated infections are a significant cause of morbidity and mortality, with postoperative surgical site infections being the second most common [2,3]. Even in the modern day, hospital infections remain a major public health concern. In the past several years, the role of hospital environmental contamination, in the development of these illnesses, has received a lot of attention. The environment's role in patient contamination, particularly adjacent surfaces and furniture, is a contentious issue. These surfaces serve as reservoirs for microorganisms, increasing the risk of cross-contamination through direct or indirect contact with patients [4-7].

Previous studies have offered valuable insights into the microbial contamination of OTs, with variations in findings influenced by factors such as geographic location, patient demographics, and healthcare practices [8, 9]. Understanding these variations and drawing comparisons with previous studies is essential for both assessing the uniqueness of the newly established tertiary care center’s OTs and identifying common trends in microbiological contamination that transcend geographic boundaries.

Moreover, the study aims to correlate its findings with recent recommendations from the World Health Organization (WHO) regarding preoperative measures for surgical site infection prevention [10]. The WHO's guidance serves as a benchmark for best practices in infection control, and its incorporation into the discussion highlights the study's commitment to aligning with the most current and globally recognized standards.

Microbiological surveillance of OTs is a critical aspect of healthcare operations, ensuring that these essential facilities remain aseptic and safe for surgical interventions. By examining the microbial landscape in the context of a newly established tertiary care center, this study aims to provide valuable insights for healthcare practitioners and policymakers, ultimately contributing to enhanced patient care and safety.

## Materials and methods

Study design

This study was conducted in the Department of Microbiology, All India Institute of Medical Sciences (AIIMS), Rae Bareli, India. Institutional Ethical Committee approval was taken (IEC-2023-10-OTH-EXP-5).

Inclusion criteria

Under the appropriate sterile conditions, samples were taken on Monday from every OT.

Exclusion criteria

The samples from the OT were not collected if it was opened before 12 hours. Additionally, the samples were rejected if contamination occurred during collection or transportation.

Data collection

The commencement of OTs began in October 2021 and continued until December 2022. Monitoring started as the OTs were microbiologically declared competent, and the data included in this study extend up to July 2023.

The samples were collected for a period of one year from nine OTs. The division of nine OTs was as follows: OT1: Neurosurgery, OT2: Pediatric Surgery, OT3: General Surgery, OT4: Urology, OT5: Orthopedics, OT6: CTVS, OT7: Ophthalmology, OT8: ENT, OT9: Obstetrics and Gynecology. OTs were cleaned with soap and water, disinfected, and fogged with quaternary ammonium compounds. After sealing OTs overnight, samples were collected aseptically. Six surfaces, namely, the floor, wall, table, light, anesthesia workstation, and door handle, were swabbed. Air was sampled via the settle plate method, and surfaces were sampled using swabs. Samples were appropriately labeled and transported immediately to the institution's microbiology laboratory.

Culture

Swabs collected from various locations were inoculated on blood agar and MacConkey agar and incubated aerobically for 18 to 24 hours at 37°C. Following an aerobic 18-24 hour incubation period of blood agar (for the air sample) at 37°C, the colonies were enumerated and translated into colony-forming units per cubic meter (CFU/m^3^) of air using the Omeliansky formula [11].

*N* = 5*a* × 10^4^ (*bt*)^−1^

where *N* is the colony-forming unit per cubic meter of air (CFU/m^3^), *a* is the number of colonies per petri dish, *b* is the surface area of the petri dish in cm^2^, and *t* is the time exposure (minutes).

Microscopy

A smear from a bacterial colony was made, and Gram's staining was done and observed under oil immersion. The colony shape, motility, catalase, coagulase, oxidase, and several biochemical tests were used to identify the isolates.

Statistical analysis

IBM SPSS Statistics for Windows, Version 20 (Released 2011; IBM Corp., Armonk, New York) was used for analysis of the data. The chi-square test was used for qualitative data whereas unpaired t-tests were used for quantitative data. P-value <0.05 was considered statistically significant.

## Results

During OT commencement, 247 swabs from nine OTs yielded 19 (7.69%) positives for bacterial growth. These microorganisms were primarily non-pathogenic, including aerobic spore-forming bacilli and micrococcus, with an average bioload of 9.5 colony-forming units (CFU/m^3^) of air (Table 1).

**Table 1:** Commencement: surface sampling and air bioload of OTs based on sterility and culture positivity (non-pathogen vs pathogen). OT: operation theater, ASB: aerobic spore-forming bacilli, CFU/m^3^: colony-forming unit per cubic meter.

OT	Total swabs	Number of sterile swabs	Number of positive cultures	Percentage of non-pathogens in positive cultures	Percentage of pathogens in positive cultures	Air bioload (CFU/m^3^)
1	26	26	0	0	0	14
2	51	47	4	25% ASB	75% *Aspergillus* spp.	13
3	12	11	1	100% Micrococcus	0	1
4	13	12	1	0	100% *Candida* spp.	1
5	37	29	8	50% Micrococcus + 12.5% ASB	12.5% MRSA + 25% MSSA	11
6	24	24	0	0	0	10
7	34	31	3	100% ASB	0	14
8	27	26	1	100% Micrococcus	0	12
9	23	22	1	100% ASB	0	10
Total	247	229	19 (7.69%)	63.15%	7 (36.84%)	Average: 9.5

During OT monitoring, swab positivity was 10.79% (23/213). The General Surgery OT showed the highest bacterial growth (5 out of 27). Surface sampling revealed prevalent 39.13% MRCoNS (methicillin-resistant coagulase-negative staphylococci), followed by 17.39% MSSA (methicillin-sensitive *Staphylococcus aureus*), MSCoNS (methicillin-sensitive coagulase-negative staphylococci), and aerobic spore-bearing bacilli at 13.04%. The differences between pathogens and non-pathogens were statistically significant. The average air bioload during monitoring of all OTs ranged between 6 and 53 CFU/m^3^. The highest air bioload was found in General Surgery, OBGYN, ENT, and Pediatric Surgery OTs at 53, 49, 47, and 42 CFU/m^3^ respectively (Table 2).

**Table 2:** Monitoring: surface sampling and air bioload of OTs based on sterility and culture positivity (non-pathogen vs pathogen). OT: operation theater, CFU/m^3^: colony forming unit per cubic meter, MRCoNS: methicillin-resistant coagulase-negative staphylococci, MSSA: methicillin-sensitive *Staphylococcus aureus*, MSCoNS: methicillin-sensitive coagulase-negative staphylococci, CTVS: Cardiothoracic and Vascular Surgery, ENT: Ear Nose Throat, OBGYN: Obstetrics and Gynecology.

OT	Total swabs	Number of positive cultures	P-value	Percentage of non-pathogen in positive cultures	Percentage of pathogens in positive cultures	Air bioload (CFU/m^3^)
Neurosurgery	20	1	0.034	0	100% MRCoNS	12
Paediatric Surgery	25	2		0	50% MSSA + 50% *Acinetobacter* spp.	42
General Surgery	27	5		20% ASB	40% MRCoNS + 20% *Pseudomonas* spp. + 20% *E. coli*)	53
Urology	21	2		0	100% MRCoNS	6
Orthopedics	23	2		0	50% MRSA+ 50% MSSA	36
CTVS	23	1		100% ASB	0	21
Ophthalmology	24	1		0	100% MRCoNS	25
ENT	24	4		25% ASB	75% MSCoNS	47
OBGYN OT	26	5		0	60% MRCoNS + 40% MSSA	49
Total	213	23 (10.79%)		3 (13.04%)	20 (86.95%)	Average: 32.33

During monitoring, among the various surfaces of OTs, OT tables were highly contaminated, followed by walls, although the difference was not statistically significant (Table 3).

**Table 3:** Monitoring: contamination status of surfaces of OTs. OT: operation theater, ASB: aerobic spore-forming bacilli, MSCoNS: methicillin-sensitive coagulase-negative staphylococci, MRCoNS: methicillin-resistant coagulase-negative staphylococci.

S. No.	Surfaces	Number of sterile swabs	Number of positive cultures	P-value	Percentage of non-pathogens in positive cultures	Percentage of pathogens in positive cultures
1	Floor (22)	18	4	0.0937	25% ASB	75% MSCoNS
2	Wall (46)	41	5		0	60% MRCoNS + 40% MSSA
3	Table (23)	14	9		11.11% ASB	44.4% MRCoNS + 11.1% *Pseudomonas* spp. + 11.1% *E. coli* + 22.2% MSSA
4	Light (23)	21	2		0	50% MRSA + 50% *Acinetobacter* spp.
5	Door handle (22)	20	2		50% ASB	50% MRCoNS
6	Anesthesia workstation (15)	14	1		0	100% MRCoNS
7	Total (213)	190	23		3 (13%)	20 (87%)

The average bioload during commencement was 9.5 CFU/m^3^ of air, while during monitoring it was 32.33 CFU/m^3^, and the difference between the two was found to be statistically significant (Table 4).

**Table 4:** Commencement and monitoring: comparison of air bioload of OTs. Here, the commencement and monitoring bioload difference is statistically significant as the p-value is 0.005. OT: operation theatre, CFU/m^3^: colony forming unit per cubic meter, CTVS: Cardiothoracic and Vascular Surgery, ENT: Ear Nose Throat, OBGY: Obstetrics and Gynecology.

OT No.	Commencement: Air bioload (CFU/m^3^)	OT Name	Monitoring: Air bioload (CFU/m^3^)
1	14	Neurosurgery	12
2	13	Paediatric Surgery	42
3	1	General Surgery	53
4	1	Urology	6
5	11	Orthopedics	36
6	10	CTVS	21
7	14	Ophthalmology	25
8	12	ENT	47
9	10	OBGY OT	49
Average	9.5	Average	32.33

## Discussion

Patients and their families may suffer severe consequences if an OT is contaminated by microbes, resulting in postoperative illnesses. Suspected hospital-acquired infections (HAIs) are investigated by collecting cultures from patients, staff, and the environment [12]. Meaningful data can only be obtained by carefully selecting the specimens to be cultivated. Long-term disability, increased resistance to antibiotics, extended hospital stays, unnecessary mortality, and a significant financial burden on health systems are all consequences of infections. Thus, a well-executed infection control program can raise staff awareness and accountability while simultaneously conducting research to modify and evaluate surveillance procedures in light of the realities of developing nations to attain satisfactory results. This can reduce the incidence of HAIs by around one-third [13]. The environmental cleaning and instrument sterilization processes undoubtedly require the most careful observation out of all the procedures and guidelines.

During OT commencement, 247 swabs from nine OTs yielded 19 (7.69%) positives for bacterial growth. These microorganisms were primarily non-pathogenic, including aerobic spore-forming bacilli and micrococcus, with an average bioload of 9.5 colony-forming units (CFU)/m^3^ of air. During OT monitoring, swab positivity was 10.79% (23/213). The bacterial count was highest in the General Surgery OT (5/23), Obstetrics and Gynecology OT (5/23) followed by the ENT OT (4/23). With bacterial counts of 1/23 each, the Neurosurgery, Ophthalmology, and Cardiothoracic and Vascular Surgery (CTVS) OTs were the least contaminated. The most common isolates during commencement were aerobic spore-forming bacilli and Micrococcus, while during monitoring, the commonest isolates were MRCoNS (9/23) 39.13% followed by MSSA (4/23) 17.39%, and MSCoNS and ASB (3/23) 13.04%.

It is interesting to note that Najotra et al. collected 4378 samples, with 195 (4.4%) being contaminated with pathogenic and non-pathogenic bacteria. *Bacillus* (184) and coagulase-negative staphylococci (17) were the most common isolates. Air sampling of several OTs revealed air bioloads in ranges of 27-133 CFU/m^3^, with the least amount of contamination in the ophthalmic OT and the highest rate of contamination in the general surgery OT, despite the fact that their study surfaces were less infected [14].

Shukla et al. found that 29.7% of 1640 swab samples in eight OTs were positive for bacterial contamination, with most being non-pathogenic bacteria like Micrococcus and aerobic spore-forming bacilli. Air bioload varied across different operating theaters, with the highest contamination in the General Surgery OT [15].

Kausar et al. found positive swabs and air samples, with *Bacillus* being the most predominant microorganism. The average air bioload ranged from 4.4 to 268.7 CFU/m^3^, with the lowest in ophthalmology and the highest in gynecology and obstetrics. *Bacillus* was the most common microbe, followed by coagulase-negative staphylococci [16].

Kiranmai and Madhavi's study identified 48 distinct bacterial species from 111 swab samples from all OTs and ICUs, with OT tables showing the highest contamination level, similar to our research. The study found that OTs had a bacterial CFU rate of 6 to 72 CFU/m^3^, with *Bacillus* species (36, 75%) and *Micrococcus* species (17) being the most frequently isolated species [17].

Deepa et al. conducted a study on microbiological flora in critical care units, revealing that bioload helps monitor air filter effectiveness, assess quality, and adjust measures for maintaining air quality. They emphasized the importance of strengthening surveillance and laboratory capacity for improved infection prevention and control [18].

The WHO has recommended preoperative measures for surgical site infection prevention, including discontinuing hair removal inside the operating room and ensuring patients are bathed with soap and water [19].

The study suggests that general surgery OTs should be cleaned and sterilized more frequently and meticulously due to the high patient load. Reducing unnecessary traffic and ensuring adequate ventilation are also crucial. Bacterial air count, a costly indicator for predicting postoperative infections, should be used to predict infections.

The National Infection Control Guidelines, Draft version 2020, recommend preventing and managing infections in Operating Rooms (OTs). Housekeeping surfaces should be cleaned daily or more frequently in high-risk areas, such as ICUs, transplant units, isolation rooms, burns wards, OTs, emergency rooms, and areas with known transmissible infectious diseases. OTs should be thoroughly cleaned once a week, including furniture, lights, equipment, windowsills, ledges, scrub rooms, and sinks [20].

A major limitation of this study was the use of coarse methods, such as the settle plate method for air sampling and the swabbing technique for surface sampling.

## Conclusions

Microbiological surveillance in OTs ensures sterility. Distinct microbial types were observed during OT commencement and monitoring phases. In newly constructed non-operational OTs, non-pathogenic organisms prevailed. However, as the OTs became functional, pathogenic organisms became more prevalent. Sampling emphasized contamination in areas with high patient loads, such as General Surgery, Obstetrics and Gynecology, and ENT OTs. Notably, OT walls and tables exhibited higher pathogenic microorganism contamination. By combining both initial commencement and ongoing monitoring, the institution has effectively managed the microbial environment within its OTs.
